# Significant Biogenic Source of Oxygenated Volatile Organic Compounds and the Impacts on Photochemistry at a Regional Background Site in South China

**DOI:** 10.1021/acs.est.4c05656

**Published:** 2024-11-01

**Authors:** Xiaopu Lyu, Hongyong Li, Shun-Cheng Lee, Enyu Xiong, Hai Guo, Tao Wang, Joost de Gouw

**Affiliations:** † Department of Geography, Hong Kong Baptist University, Hong Kong 999077, China; ‡ Thrust of Earth, Ocean and Atmospheric Sciences, Hong Kong University of Science and Technology (Guangzhou), Guangzhou 511455, China; § Thrust of Sustainable Energy and Environment Hong Kong University of Science and Technology (Guangzhou), Guangzhou 511455, China; ∥ Department of Civil and Environmental Engineering, The Hong Kong Polytechnic University, Hong Kong 999077, China; ⊥ School of Environmental Science and Engineering, Southern University of Science and Technology, Shenzhen 518055, China; # Cooperative Institute for Research in Environmental Sciences & Department of Chemistry, University of Colorado Boulder, Boulder, Colorado 80309, United States

**Keywords:** oxygenated volatile organic compounds, ozone pollution, biogenic emissions, acetaldehyde, photochemistry

## Abstract

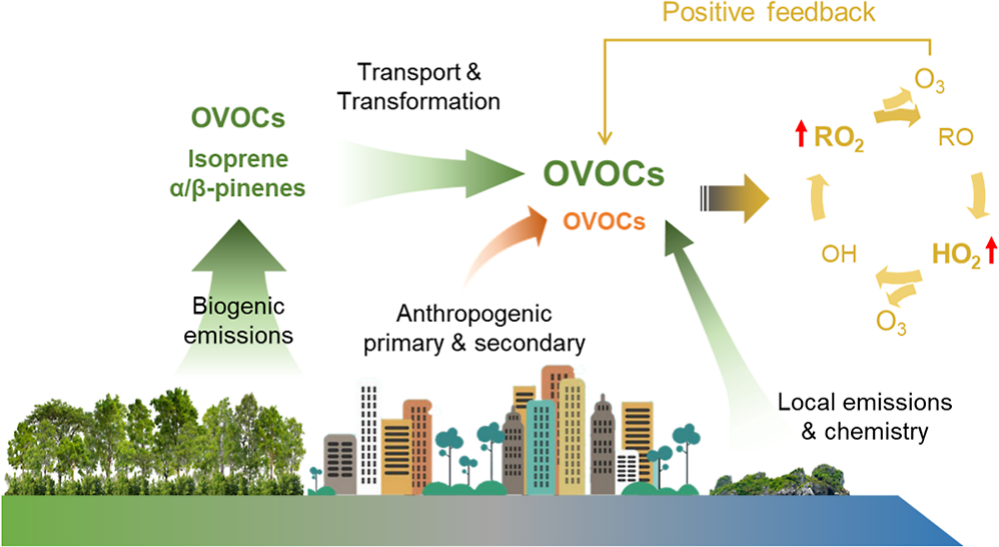

Oxygenated volatile organic compounds (OVOCs) significantly modulate atmospheric chemistry, but the sources and air quality impacts of OVOCs in aged urban outflows remain to be elucidated. At a background site in South China, the ozone formation potential of six nonformaldehyde OVOCs studied was equivalent to that of 3.56 ppbv of formaldehyde, more than half of which was contributed by acetaldehyde. Source apportionment incorporating photochemical age revealed that considerable fractions (52.7%–62.6%) of the OVOCs were of biogenic origin, except for ethanol, which was primarily derived from anthropogenic emissions. The oxidation of *cis*-/*trans*-2-butene explained 71.1% of the in situ acetaldehyde formation. In contrast, α/β-pinenes and isoprene contributed 73.8% and 28.4% to acetone and methylglyoxal formation, respectively. An average of 12.4% of net in situ ozone (O_3_) production rate was attributed to the OVOCs studied, where the biogenic fractions accounted for 59%. The changes in the O_3_ production rate and hydroxyl radical (OH) concentration caused by OVOCs were mainly affected by ozone formation sensitivity. The effects of primary acetaldehyde and acetaldehyde-led O_3_ on secondary acetaldehyde formation were weak at this background site; however, they cannot be ignored in polluted areas. This study provides a scientific basis for mitigating O_3_ pollution driven by biogenic emissions and OVOCs.

## Abstract 2

In situ O_3_ formation and radical chemistry at a regional background site were markedly impacted by OVOCs that were primary derived from biogenic emissions and/or transformation.

## 1 Introduction

China faces increasingly severe challenges of ground-level ozone (O_3_) pollution, which has been exacerbated by uncoordinated emission reduction of the precursors, especially nitrogen oxides (NO_*x*_) and volatile organic compounds (VOCs).^1,2^ Oxygenated VOCs (OVOCs) are of wide concern due to their contributions to atmospheric radicals, O_3_, and secondary particulate matter,^3−5^ as well as the potential health risks of exposure to some of them.^6^ For example, the OVOCs, built up primarily through VOC oxidation, were confirmed to serve as the dominant source of radicals and led to a week-long winter O_3_ pollution event in an oil and gas basin in the United States.^7^ OVOCs can be emitted from various sources, such as terrestrial vegetation, fossil fuel combustion, and oceanic emissions, and can also be formed through the oxidation of hydrocarbons.^8,9^ Insufficient understanding of these processes can lead to missing sources of OVOCs, such as that discovered over the tropical Pacific in the early 2000s.^8^

Quantitative analysis of OVOC sources has been a research hotspot. Apportionment methods based on multiple linear regression (MLR), emission statistics and emission-based modeling, and receptor models are commonly used.^10−12^ As elucidated by Parrish et al., correlation-based source apportionment of OVOCs is subject to errors due to reactivity differences between the species involved.^10^ This applies to MLR and positive matrix factorization analysis with primary and secondary air pollutants as tracers. To address this issue, De Gouw et al. first proposed a parametrization method incorporating photochemical aging.^13^ The method is extensively used where the required data are available, e.g., methacrolein (MACR) and methyl vinyl ketone (MVK) to correct for chemical loss of isoprene.^14,15^

Biogenic emissions and related chemistry are an important source of OVOCs.^16−18^ Methanol, acetaldehyde, and acetone were identified as the most abundant OVOCs at a hardwood forest.^16^ Biogenic source, including primary and secondary fraction, was shown to be the largest source of formaldehyde and acetaldehyde in urban areas of a North China city (Beijing) and a South China city (Shenzhen).^17^ The oxidation of biogenic VOCs, mainly isoprene and acetone, accounted for over 80% of methylglyoxal (MGLYOX) production globally.^18^ Photolysis of OVOCs produces oxidative radicals and impacts atmospheric chemistry, making potentially considerable contributions to the production of O_3_. OVOCs were shown to be the largest source of radicals at a rural site in the North China Plain, the neglect of which would lead to biased understanding of O_3_ formation sensitivity.^3^ At an urban site in Guangzhou, South China, radical production from nonformaldehyde OVOCs was even higher than that produced by formaldehyde and nitrous acid.^4^

Despite the previous work, there are remaining areas where the knowledge of OVOCs is not well-defined. First, the importance of biogenic OVOCs in urban air outflows, which are considered to be rich in anthropogenic pollutants, has not been clearly evaluated. Second, OVOC formation can be mediated by radical precursors, like primary OVOCs and O_3_, but it has been rarely studied. Comprehensive measurements of secondary air pollution at a regional background site in Hong Kong (HK) allowed us to explore the above issues. In this study, we focus on the sources of six nonformaldehyde OVOCs, i.e., acetaldehyde, acetone, acrolein, MGLYOX, methanol, and ethanol, and their roles in mediating in situ photochemistry. The species were selected based on their concentrations, data quality, and difference in reactivity (Text S1). Although OVOCs have been extensively studied in HK,^19,20^ this is the first attempt to determine the source contributions to OVOCs by incorporating photochemical age and to reveal photochemical interactions between primary and secondary fractions of OVOCs and O_3_ using a near-explicit chemical mechanism with full constraints. We show that biogenic emissions and chemistry dominate the sources of the OVOCs studied. Correctly attributing this source is essential for the design of air quality management.

## 2 Methodology

### 2.1 Sampling Campaign

In autumn 2020, a joint field campaign with the theme of secondary air pollution observation was conducted at a regional background site in South China.^21,22^ The Hok Tsui site is an air quality monitoring station on the coastline of the South China Sea and is operated by the HK Environmental Protection Department (EPD). In general, it receives continental outflows in autumn, which was confirmed by the 72 h backward trajectories on the selected dates we focus on in this study, as shown in Figure S1. The air masses reaching the site passed over southeastern China with high vegetation coverage, as indicated by the leaf area index (LAI) in autumn 2020. Besides, HK has a vegetation coverage rate of ∼70%, and the LAI close to the site was also high.

In addition to the regular measurements conducted by the EPD, a set of state-of-the-art instruments were temporarily deployed at the site to measure organic compounds in gas and condensed phases, radicals, and other key species/parameters (e.g., nitrous acid). Table S1 lists their measurements and uses in this study. OVOCs and dozens of other VOCs with a higher proton affinity than water were measured by an Ionicon proton transfer reaction quadrupole ion time-of-flight mass spectrometer (PTR-ToF-MS, referred to as PTR hereinafter), which has been described elsewhere.^23^ Formaldehyde measurement was not fully calibrated, and the humidity interference cannot be eliminated, so the data are not used in this study. In addition to the online instruments, 50 whole air samples were collected with precleaned and vacuumed stainless steel canisters on 10 selected dates. The sample collection started at 9:00 local time (LT) and ended at 18:00 LT at odd hours every day, with the duration of 1 h for each sample. The samples were analyzed within 1 week using gas chromatography (GC) coupled with mass spectrometry, flame ionization detection, and electron capture detection. The concentrations of 79 VOC species were quantified. More details about the analysis methods can be found in previous papers.^24^Table S2 lists the metrics of data quality control for all the VOCs and OVOCs used in this study. Because we use this set of chemically comprehensive VOC data in photochemistry modeling, our analysis mainly focuses on the 10 dates when offline VOC data was available.

Interferences with PTR measurements are common for some species, including acetaldehyde and isoprene that are relevant to this study. The reaction of ethanol with O_2_^+^ produces a signal at *m*/*z* 45, interfering with acetaldehyde measurement. However, Ionicon PTR has low levels of O_2_^+^ due to the separation between the ion source and the sampled air.^25^ Moreover, the average concentration of *m*/*z* 47 (ethanol, 0.33 ppbv) was much lower than that of *m*/*z* 45 (acetaldehyde, 2.14 ppbv). Given the ratio of byproduct *m*/*z* 45 to ethanol determined by previous studies (0.22–0.38),^25,26^ the ethanol interference to acetaldehyde was estimated to be lower than 6%. Positive bias often occurs for PTR measurements of isoprene, especially in urban areas, due to the fragmentation of long-chain aldehydes and cycloalkanes to *m*/*z*69. Coggon et al.^25^ proposed a correction method based on the ratio of *m*/*z* 69 to the sum of *m*/*z* 111 and *m*/*z* 125 in a nighttime period when biogenic emission of isoprene was assumed to be as low as zero. The logic behind this was that *m*/*z* 111 and *m*/*z* 125 were generated simultaneously with *m*/*z* 69 by the interfering substances (e.g., long-chain aldehydes from cooking) but are not the product ions of isoprene. In this study, *m*/*z* 125 was not detected and isoprene was corrected using the ratio of *m*/*z* 69 to *m*/*z* 111, as described in Text S2. Figure S2 shows the observed *m*/*z* 69, corrected *m*/*z* 69 and isoprene measured by the online and offline GC systems (Table S1). The correction improved the agreement between the PTR and GC measurements, particularly for the pair of PTR and offline GC data (*R* = 0.94). Due to the missing data at *m*/*z* 111, 29.6% of the hourly isoprene data was not corrected. We use a combined data set of original PTR isoprene and corrected isoprene for the following analysis.

### 2.2 Parameterization of OVOC Sources

To resolve the sources of OVOCs, we adopt the nonlinear parametrization method developed by De Gouw et al. that considers primary fraction loss and secondary fraction formation of OVOCs during photochemical aging.^13^ The observed concentration of a specific OVOC, denoted [OVOC], is regarded as the sum of four components: anthropogenic primary emission, anthropogenic secondary formation, biogenic origin, and background, as described in Formula 1.
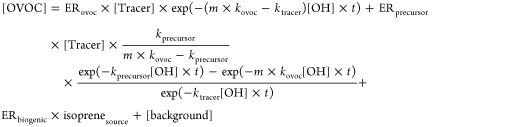
1

**Figure 1:**
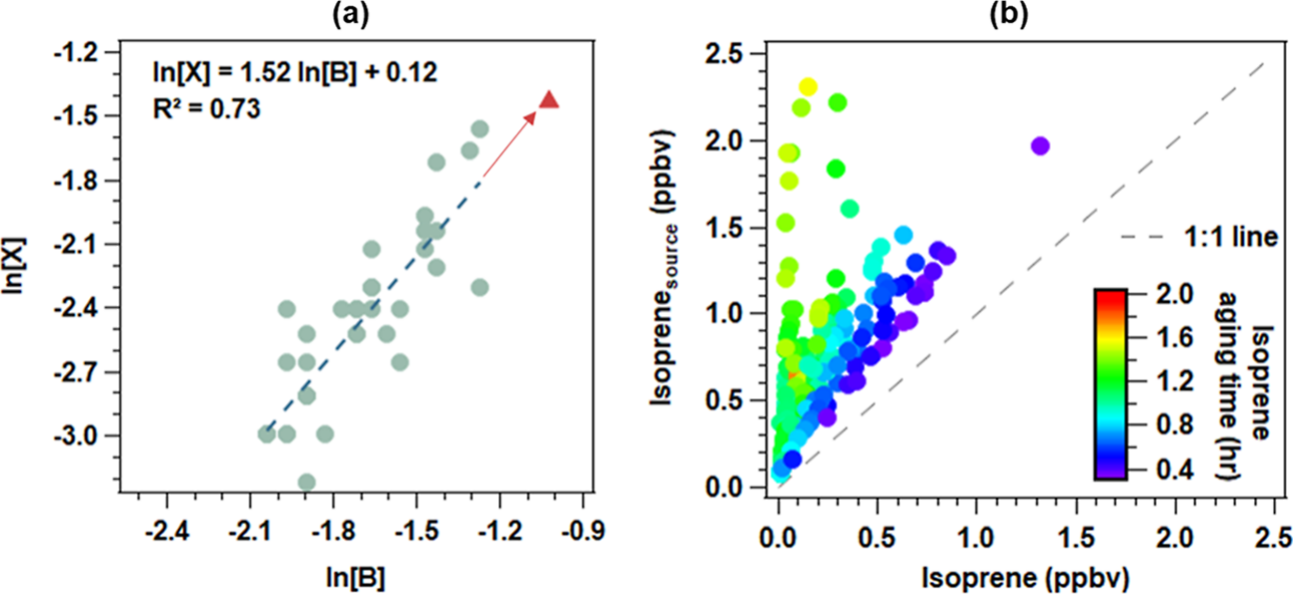
Linear regression of natural log xylenes (ln[X]) and benzene (ln[B]) during 18:00–0:00 (a); scatter plot of isoprene_source_ versus isoprene color-coded by isoprene aging time (b). Red triangle in (a) represents the point with the highest level of benzene.

[Tracer] is the concentration of the anthropogenic tracer benzene in this study. Benzene correlated moderately with acetylene (*R* = 0.70) and carbon monoxide (CO, *R* = 0.85), the other two tracers commonly used in this method. [OH] × *t* stands for hydroxyl radical (OH) exposure, calculated using Formula 2. The initial *m*,*p*-xylenes (X) to benzene (B) ratio with the photochemical age *t* of 0, , is critical for the OH exposure calculations. It was calculated in the same way as that used in a previous study (see Text S3).^14^ In brief, we performed a linear fit between the natural log of xylenes and benzene during 18:00–0:00 when the X/B ratios were the highest, extrapolated the regression line to the highest benzene, and calculated the ratio of the fitted xylenes to benzene at this point, as shown in Figure 1a. The  is estimated to be 0.66, which was between the third and fourth highest X/B ratio in the study period. It was also close to the X/B ratio measured in a tunnel (0.61 ± 0.17)^27^ and at five roadside sites in HK (0.73 ± 0.13) (Table S3).
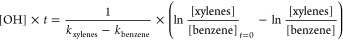
2

ER_ovoc_ and ER_precursor_ in Formula 1 are the emission ratios of OVOC and its precursors to the anthropogenic tracer, respectively. ER_biogenic_ represents the ratio of biogenic OVOC to isoprene_source_, the isoprene corrected for chemical loss. All the *k*_X_ are reaction rate constants of the species X with OH, obtained from the Master Chemical Mechanism (MCM) v3.3.1 and Atkinson and Arey.^28^ The coefficient *m* in Formula 1 is a correction factor that accounts for the loss of OVOC through photolysis. It was determined to be 1.19 for acetone, 2.68 for MGLYOX, and 1 for the others with very weak photolysis compared to OH-initiated oxidation based on the simulated rates of OVOC loss pathways at this site (see Text S4). ER_ovoc_, ER_precursor_, ER_biogenic_, *k*_precursor_, and [background] are unknown values and determined from the least-squares fit. The isoprene_source_ was calculated based on the ratio of *m*/*z* 71 (sum of MACR and MVK) to isoprene that indicates the aging time of isoprene, as illustrated in De Gouw et al.^13^Figure 1b shows the scatter plot of isoprene_source_ and isoprene color-coded by the isoprene aging time. It is noteworthy that the isoprene aging time differed significantly in magnitude and pattern from the photochemical age determined from the X/B ratios. Therefore, it is recommended not to use the ratio of biogenic tracers to calculate the OH exposure that influences the loss and formation of anthropogenic OVOCs in this method.

### 2.3 In Situ Photochemistry Modeling

The Framework for 0-D Atmospheric Modeling (F0AM) nested with MCM v3.3.1 (https://mcm.york.ac.uk/MCM) was employed to simulate the in situ photochemistry. The model was constrained by observed air pollutants and meteorological parameters every 10 min, as listed in Table S4. Formaldehyde measurements were not used in the simulations. The data with higher time resolutions, such as OVOC and trace gas data, were converted to 10 min averages. We used the linear interpolation to derive the 10 min VOC data in daytime from the discontinuous data (data points at every 2 h interval). Offline VOC samples were not collected at night, so nocturnal profiles were not available. However, they had little impact on observation-based simulation of photochemistry and were estimated based on the daytime averages and diurnal profiles of similar species measured by the online GC and PTR. While NO_*x*_ was constrained to the observed values, the partitioning between nitric oxide (NO) and nitrogen dioxide (NO_2_) was allowed to evolve over time. The photolysis frequency of NO_2_ (jNO_2_) was input into the model, and the ratio of measured jNO_2_ to simulated jNO_2_ was used to correct the photolysis frequencies of the other species. For the simulation of a specific day, the model was run without interruption for 5 days by using the same diurnal profiles of input. The first 4 days of simulation were treated as model spin-up, and the output of the last day was taken. The model performance was evaluated by comparing the simulated and observed OH, whose measurement was detailed in another paper.^21^Figure S3 shows the time series of simulated and observed OH, NO, NO_2_, and NO/NO_2_ ratio. The model reproduced the levels and variations of these highly reactive species reasonably well, except that the OH was overestimated, and the NO/NO_2_ ratio was significantly underestimated on 4 November. Constraining the NO/NO_2_ ratio to observed values did not improve the OH simulation. Sensitivity tests indicated a missing OH sink on this day, which was identified in other cases by a previous study and was suspected to be related to unmeasured trace gases.^21^ Since it is difficult to account for this missing sink in the model, we excluded the simulation results on 4 November from the in situ photochemistry analysis. The in situ net ozone production rate (OPR) was calculated according to Formula 3(24)
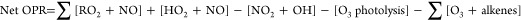
3where inside the square brackets are reactions, and the calculation is based on reaction rates. HO_2_ and RO_2_ are hydroperoxyl radicals and a collection of alkylperoxyl radicals, respectively.

The reaction rate Ratio of Nitric acid formation from NO_2_ + OH to the sum of Self-reactions between peroxyl radicals is defined as NSR and calculated as per Formula 4.
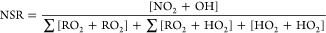
4

Based on the existing understanding of chain termination reactions under NO_*x*_-rich and NO_*x*_-deficient conditions,^29,30^ higher (lower) NSR implies a more VOC-limited (more NO_*x*_-limited) O_3_ formation regime.

## 3 Results and Discussion

### 3.1 Overview of OVOC Data

Averaged over the selected dates, methanol was the most abundant OVOC with an average mixing ratio of 6.25 ± 0.41 ppbv, followed by acetone (4.20 ± 0.26 ppbv), acetaldehyde (2.14 ± 0.16 ppbv), ethanol (0.33 ± 0.02 ppbv), acrolein (0.29 ± 0.02 ppbv), and MGLYOX (0.12 ± 0.01 ppbv). Given its high reactivity, acetaldehyde led the contributions to ozone formation potential (OFP) (7.95 ppbv of O_3_). MGLYOX and methanol, with the OFP of 2.78 and 1.62 ppbv of O_3_, respectively, ranked the second and third. Notably, the total OFP of the six OVOCs, 54% of which was attributed to acetaldehyde, was equivalent to that of 3.56 ppbv of formaldehyde. This was much higher than the average level of formaldehyde (2.34 ppbv) in 16 daily samples collected in October–November 2020 and analyzed by the EPD (Figure S4). Acetaldehyde was highly correlated with isoprene_source_ (*R* = 0.83), and the *R* was 0.77–0.84 for the other nonethanol OVOCs (Figure S5). However, the correlation between ethanol and isoprene_source_ was much weaker (*R* = 0.58), in contrast to the correlation of ethanol with CO (*R* = 0.81), a common tracer of anthropogenic emissions (Figure S5). These correlations implied a strong link between the OVOCs other than ethanol and biogenic emissions and chemistry.

Figure S6 shows the diurnal variations of hourly mean and median levels of OVOCs, with obvious enhancements in the daytime. Except for ethanol, which had higher concentrations in the morning and early evening, the other OVOCs exhibited peaks in hourly mean values during the midday and afternoon. However, the afternoon rise was not evident in the hourly medians. The difference was mainly due to the noticeably enhanced transport of inland air to the site as the severe tropical storm Atsani approached in the afternoon of 6 November. This resulted in increased concentrations of OVOCs and many other air pollutants, both primary and secondary. Meanwhile, the sum of MACR and MVK also got the highest concentrations and brought the isoprene_source_ to the highest despite moderate levels of isoprene. The noon peaks of hourly medians for nonethanol OVOCs demonstrated dominant contributions of photochemical and/or biogenic sources to these species. Otherwise, the concentrations would be at the low end during the midday with the highest boundary layer.

### 3.2 Source Apportionment of OVOCs

**Figure 2:**
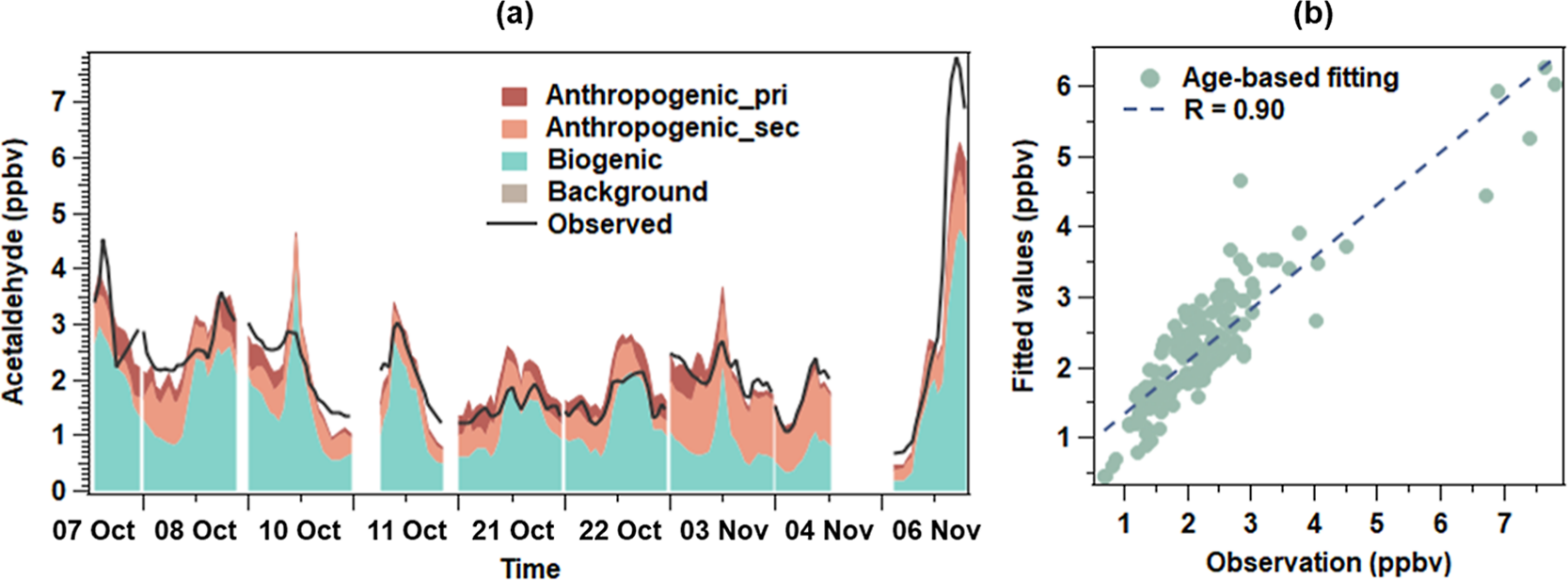
Time series of observed acetaldehyde and fitted source contributions (a); and correlation between the observed and fitted values (b). Missing data in (a) are due to unavailability of data for OVOCs, benzene, isoprene_source_, or photochemical age calculated from X/B ratios.

As described in Section 2.2, the parametrization method incorporating photochemical age was used to quantify the source contributions. The method provided reasonably good fits to the observed OVOCs, with the *R* ranging from 0.82 to 0.90. Table S5 summarizes the known and fitted values of the key parameters in this method. Negative values were set as zero, as adopted in previous studies.^13,14^Figure 2 shows the time series of the observed acetaldehyde and the fitted values attributable to the sources of anthropogenic primary emissions (anthropogenic_pri), anthropogenic secondary formation (anthropogenic_sec), and biogenic origins (biogenic). The fitted background level of the acetaldehyde was negligible. The sum of anthropogenic_pri, anthropogenic_sec, and biogenic acetaldehyde agreed well with the observed values (*R* = 0.90). Even excluding the few data points on 6 November with the mixing ratio above 6 ppbv, the *R* was still as high as 0.84. The fit correlation was much higher than the MLR (*R* = 0.56) with CO and O_3_ as the primary and secondary tracer, respectively.

Based on the fitting results, the biogenic source, including direct emissions and chemical transformation of the emissions, constituted the largest fraction (61.5%) of the observed acetaldehyde, which was almost twice the total contribution of anthropogenic_pri (11.4%) and anthropogenic_sec (27.1%). Many studies reported the primary emissions of acetaldehyde from plants under various physiological conditions. The mechanisms include at least the oxidation of ethanol produced in anoxic tissues, normal photosynthesis and transpiration, and light–dark transitions, such as light-flecks throughout the day.^31−33^ In addition, biogenic VOCs (e.g., ethanol and light alkenes) can indeed contribute to the formation of acetaldehyde, although probably not isoprene.^34−36^ However, it is unclear which processes played a more important role in building the acetaldehyde levels.

Biogenic emissions and chemistry were also the dominant sources of other OVOCs except ethanol, as shown in Table 1. The fraction of biogenic OVOCs varied between 52.7% for methanol and 62.6% for acrolein. Ethanol was mainly derived from anthropogenic primary emissions (67.7%), with the remaining 32.3% attributable to a biogenic source. To test the sensitivity of the source parametrization method, we also applied it to the sum of MACR and MVK and identified a biogenic contribution of as high as 88.7%. Excluding the data on 6 November with the highest levels of isoprene_source_ and MACR and MVK decreases the fitted biogenic contributions to the OVOCs studied by 4.1%–21.5% (Table S6). Thus, the high biogenic contributions were unlikely to be the result of methodological flaws.

**Table 1:** Source Contributions (Mean ± 95% Confidence Interval) to OVOCs and Correlation Coefficients of Fitted and Observed Values

OVOC species	*R*	anthropogenic_pri (%)	anthropogenic_sec (%)	biogenic (%)	background (%)
acetaldehyde	0.90	11.4 ± 0.6	27.1 ± 2.9	61.5 ± 0.4	0
acetone	0.85	17.9 ± 0.7	0	54.2 ± 0.4	27.9 ± 0.8
acrolein	0.86	7.8 ± 0.5	19.2 ± 5.4	62.6 ± 0.5	10.4 ± 0.9
MGLYOX	0.88	0	34.4 ± 7.3	61.7 ± 0.4	3.9 ± 0.8
methanol	0.82	31.7 ± 0.8	0	52.7 ± 0.4	15.6 ± 0.9
ethanol	0.85	67.7 ± 0.4	0	32.3 ± 0.4	0

Previous studies on VOC sources in HK did not find similar results. For example, the Positive Matrix Factorization model was used to resolve the sources of VOCs (including a large portion of OVOCs) at this site and another background site.^19,23^ The studies suggested that secondary formation was the largest source of OVOCs, while the precursors were not identified. However, the factor representing secondary formation contained high loadings of isoprene, a tracer of primary biogenic emissions.^19,23^ In these previous studies, a considerable fraction of isoprene oxidation products (MACR and MVK) were apportioned to other sources, e.g., vehicle and industrial emissions.^23^ Therefore, it was likely that the biogenic contributions to OVOCs were underestimated, illustrating the inherent shortcomings of PMF for OVOC source apportionment. The null contribution of anthropogenic_sec to acetone was also inconsistent with a previous study showing *i*-butane and *i*-butene as the main precursors of acetone.^37^ While it is unknown what VOCs contributed the most to acetone formation on the transport path of air masses, our modeling results indicated that α/β-pinenes accounted for over 73% of in situ acetone formation, which is discussed below. Thus, even if the anthropogenic secondary formation was underestimated, it was unlikely to be significant.

However, studies indeed identified considerable biogenic contributions to OVOCs at many other remote sites around the world. Huang et al. found that 30–48% of autumnal methanol, acetone and acetaldehyde in the background atmosphere of a mountainous area in South China were of biogenic origin,^38^ which was also the largest source (40%) for the summertime acetaldehyde at a rural site in the North China Plain.^39^ In a background area of the Eastern Mediterranean, local biogenic contribution explained up to 64% of the OVOCs, mainly methanol and acetone.^40^ The observation over a boreal Scots pine forest in Southern Finland revealed that methanol, acetone, and acetaldehyde were the OVOCs with the highest net fluxes in summer.^41^ While there has been overwhelming evidence for the biogenic origin of MGLYOX,^18,34^ this did not appear to be the case for acrolein. However, studies confirmed the production of acrolein from fires, biogenic secondary formation and fallen leaves.^42,43^ Therefore, the biogenic contribution to acrolein determined above might be the combined result of multiple pathways.

Overall, our results highlight the significant levels of biogenically derived OVOCs. The relative emission ratio of OVOCs with reference to isoprene, ER_biogenic_, ranged from 0.11 ± 0.01 for MGLYOX to 4.96 ± 0.30 for methanol. These ratios are at the high ends of the corresponding ranges reported in previous studies. For example, the ER_biogenic_ for methanol in urban Beijing in August–September was as low as 0.02.^14^ However, much higher values were determined in the continental outflows of northeastern United States in summer (0.44),^13^ in urban Beijing from May to June (1.92) and in urban Shenzhen in April (1.24).^17^ The fitted ER_biogenic_ for methanol in this study was even higher (4.96), but the difference was consistent with those of previous studies. A similar degree of discrepancy also exists for the other OVOCs, which might be due to the differences in type, density, and growth period of vegetation and meteorological conditions. It seems unrealistic to conclude a universally applied ER_biogenic_ to parametrize biogenic OVOCs in emission-based models. To achieve this, we recommend measuring the OVOC emissions from dominant vegetation species and characterize OVOC formation from biogenic precursors.

Additionally, anthropogenic secondary formation surpassed anthropogenic primary emission for acetaldehyde, acrolein, and MGLYOX, the three more reactive OVOCs, while there was almost no secondary acetone, methanol, or ethanol formed from anthropogenic precursors. Furthermore, the diurnal patterns of biogenic OVOCs followed the same pattern as isoprene_source_. The anthropogenic_pri was the smallest in the early afternoon, likely due to chemical losses and atmospheric mixing under the high boundary layer. Similar to the patterns of secondary air pollutants shaped by transport at other background sites, the anthropogenic_sec showed a broad peak in the daytime (Figure S7).

### 3.3 In Situ Photochemistry of OVOCs

**Figure 3:**
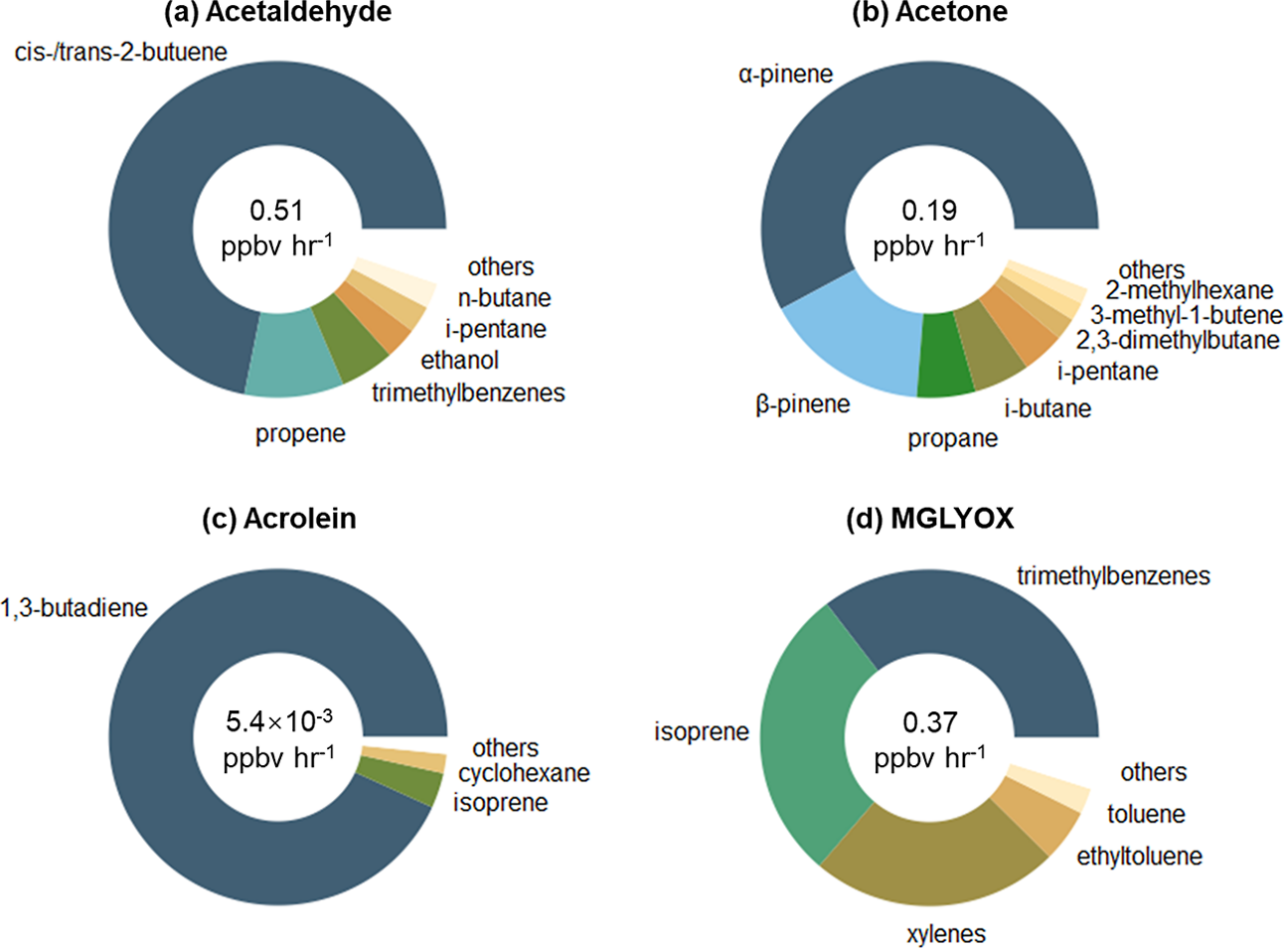
Contributions of the major precursors to the total formation rate of individual OVOCs: acetaldehyde (a); acetone (b); acrolein (c); and MGLYOX (d). Numbers in the inner cycles are the daytime average formation rates of respective OVOCs.

To understand the formation and chemical loss of OVOCs and photochemical effects, we analyzed the in situ modeling results extracted from a well-validated box model (Section 2.3). The precursors and pathways leading to the OVOC formation were first examined. Acetaldehyde, for example, experienced comparable rates of in situ formation and loss, with a relatively low net production rate of 2.69 ppbv day^–1^ (Figure S8). The loss of acetaldehyde was dominated by OH-initiated oxidation (accounting for 95.7%), 33 times faster than the photolysis loss. The formation pathways are relatively complicated. By identifying the last reactions generating acetaldehyde and tracing upward, we determined the major formation pathways and the corresponding precursors of acetaldehyde, as listed in Table S7. The identified pathways were responsible for more than 92% of the total formation rate of acetaldehyde. Among them, *cis*-/*trans*-2-butene reacting with OH, O_3_ and much less importantly nitrate radical (NO_3_) explained 71.1% of acetaldehyde formation (Figure 3). The other acetaldehyde precursors that were mainly oxidized by OH included propene, trimethylbenzenes, ethanol, *i*-pentane, and *n*-butane. Biogenic VOCs, either isoprene or α/β-pinenes, contributed little to the formation of acetaldehyde. Nevertheless, the possibility of secondary formation of acetaldehyde from biogenic precursors in the air mass history cannot be ruled out due to the dense vegetation in the northern part of the Pearl River Delta where the air masses passed. Chemical transport models with appropriate OVOC chemistry may help us understand the processes and contributions.

Similarly, we looked into the chemistry of acetone, acrolein, and MGLYOX. Methanol and ethanol were excluded from the analysis due to their minor photochemical effects. Photolysis explained 15.8%, 1.5%, and 61.3% of the loss of acetone, acrolein, and MGLYOX, respectively, and the rest were mainly attributed to OH-initiated oxidation. In terms of formation, as shown in Figure 3, acrolein was primarily derived from 1,3-butadiene, with little contributions from isoprene. In contrast, 73.8% of acetone was produced by α-/β-pinenes, and isoprene was the second largest precursor of MGLYOX, only after trimethylbenzenes. We note that α-/β-pinenes correlated fairly well with isoprene (*R* = 0.92). Thus, the biogenic acetone and MGLYOX determined by the parametrization method likely contained considerable secondary fractions.

**Figure 4:**
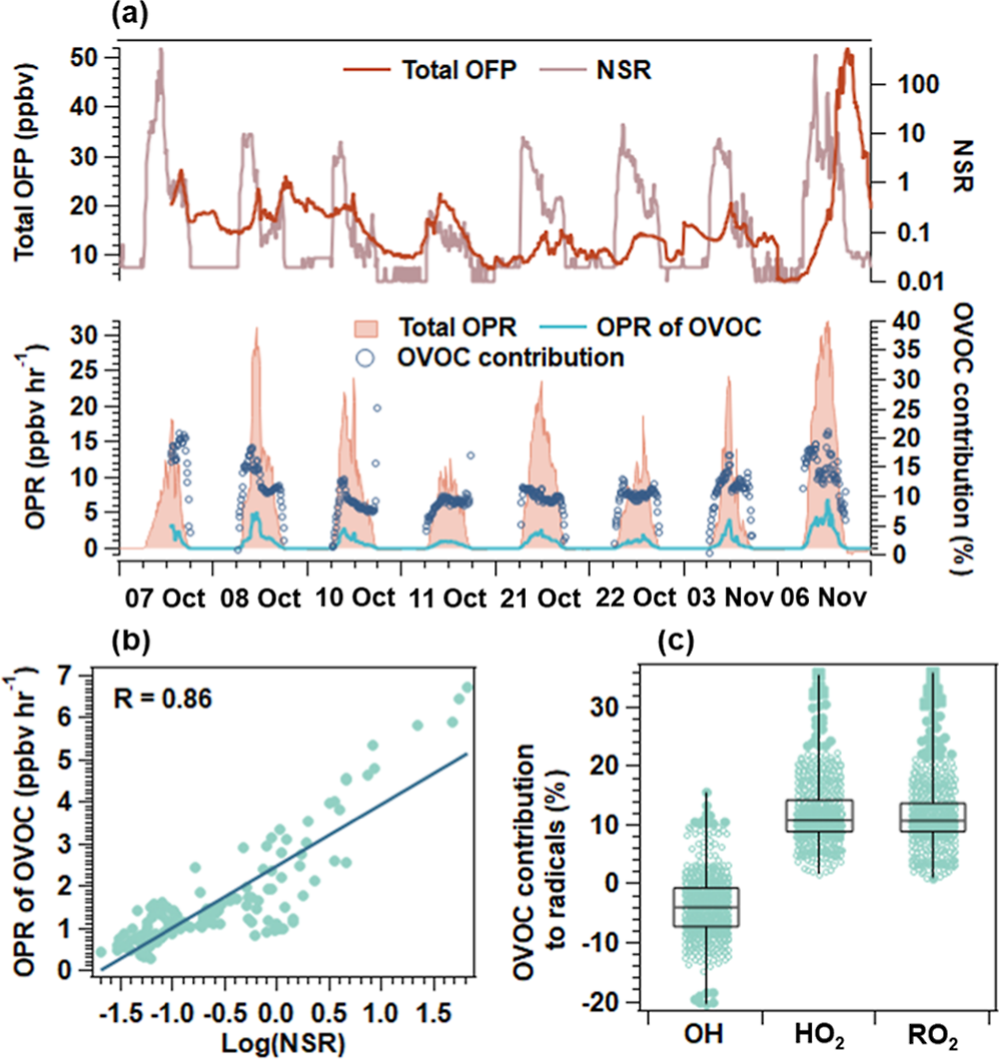
Time series of total OPR, OVOC contributions to OPR, and total OFP of OVOCs and NSR (a); correlation between OPR of OVOCs and logarithm of NSR (b); and changes in OH, HO_2_, and RO_2_ led by OVOCs (c).

The impacts of OVOCs on O_3_ and oxidative radicals were further studied. The net OPR attributed to OVOCs was 0.5 ppbv h^–1^ with the hourly maximum in the average diurnal profile of ∼2.1 ppbv h^–1^, accounting for 12.4% of the total OPR (Figure 4a). Biogenic OVOCs determined above contributed 59% to the OVOC-enhanced OPR. In the average diurnal cycle, the NSR decreased from ∼26 at 9:00 to less than 1 in the afternoon, in line with the common sensitivity evolution of O_3_ formation to the precursors in a day.^44^ The total OFP values of OVOCs were also calculated. However, the OPR contributed by OVOCs did not show any relationship with the OFP. Interestingly, it correlated moderately with the logarithm of NSR (*R* = 0.69), and the correlation was further improved during 12:00–14:00 with the *R* reaching 0.86 (Figure 4b). Therefore, it was the O_3_ formation sensitivity rather than the abundance of OVOCs that influenced the OVOC’s contribution to O_3_ production at this background site.

For the radicals, the OVOCs increased the HO_2_ and RO_2_ concentrations by 10.8% and 10.3%, respectively. Conversely, they caused an average OH reduction of 3.1% (Figure 4c). OVOC photolysis has been recognized as an important source of peroxyl radicals,^4,7^ and it was indeed responsible for all the OVOC-induced HO_2_ increase here. However, 70% of the HO_2_ increase was caused by the photolysis of a part of secondary formaldehyde that was produced by the OH-initiated oxidation of acetaldehyde (CH_3_CHO—CH_3_CO_3_—CH_3_O_2_—CH_3_O—HCHO). Hence, attention needs to be paid to the pathways other than direct photolysis when studying radical production from nonformaldehyde OVOCs. The overall reduction in OH resulting from OVOCs was also unexpected. Like the OPR of OVOCs, the change in OH was closely related to the logarithm of NSR, with the highest *R* of 0.85 during 9:00–13:00. The negative response of OH to OVOCs under low values of NSR indicated that the OH recycling via HO_2_ + NO was limited by the insufficient NO_*x*_. A similar effect was simulated in a remote mountainous area where the rate of HO_2_ reacting with NO was only 34% of the OH loss rate toward VOCs.^30^ However, the net OH reduction would not happen in a NO_*x*_-rich environment, as indicated by the OH increase under a high NSR.

### 3.4 Interactions in the OVOC–O_3_ System

**Figure 5:**
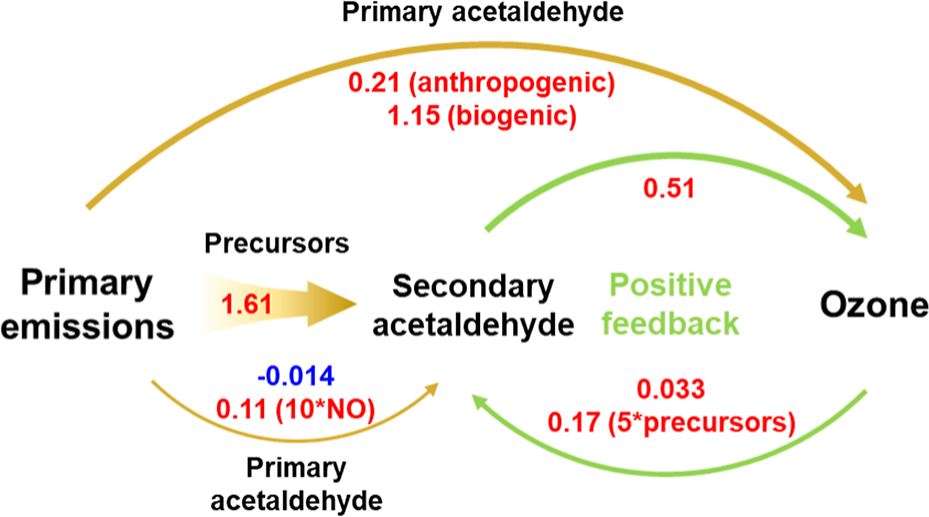
Interactions between primary acetaldehyde, secondary acetaldehyde, and acetaldehyde-led O_3_. The numbers are the simulated changes in maximum hourly OPR rate or acetaldehyde formation rate (ppbv h^–1^).

Secondary OVOCs are formed simultaneously with O_3_ through photochemical reactions. While promoting O_3_ formation, they are also affected by primary emissions (including primary OVOCs and precursors) and O_3_. In situ formation of OVOCs from the precursors were discussed above. The primary OVOCs and O_3_ influence secondary OVOC formation by providing oxidative radicals and further changing the oxidation efficiencies of OVOC precursors. Besides, O_3_ can directly react with some alkenes to produce OVOCs. For example, ozonolysis of *cis*-/*trans*-2-butene explained 31.7% of the in situ acetaldehyde formation at this site. Taking acetaldehyde as an example, we simulated the interactions in the OVOC–O_3_ system, as illustrated in Figure 5. To improve the modeling efficiency, we used the average diurnal profiles of air pollutants and meteorological parameters for the selected dates as the model input. Based on the in situ modeling results, we assume all of the biogenic acetaldehyde came from primary emissions, which presents to be an upper limit of biogenic primary acetaldehyde. With this assumption, the primary acetaldehyde (anthropogenic_pri + biogenic) increased the maximum hourly OPR at noon by 1.36 ppbv h^–1^. The enhancement of the maximum hourly OPR by secondary anthropogenic acetaldehyde was 0.51 ppbv h^–1^. In turn, the part of the O_3_ generated from acetaldehyde facilitated acetaldehyde formation at a maximum hourly rate of 0.033 ppbv h^–1^ through contributing to OH and directly reacting with *cis*-/*trans*-2-butene. In comparison, the primary acetaldehyde slightly weakened acetaldehyde formation by −0.014 ppbv h^–1^. This negative effect was due to the OH consumption by acetaldehyde and inefficient OH recycling limited by NO_*x*_, as stated above. It is worth noting that the role of additional acetaldehyde in sequestering NO_*x*_ into peroxyacetyl nitrate was not considered in the simulations due to NO_*x*_ constraint.

The simulated effects of primary acetaldehyde and acetaldehyde-led O_3_ on secondary acetaldehyde formation were weak. However, it does not necessarily mean that the interaction mechanisms are negligible in any cases. Sensitivity tests indicated that the weak effects were due to the low levels of OVOC precursors and NO_*x*_ at this site. The positive feedback of acetaldehyde-led O_3_ to secondary acetaldehyde formation was proportional to the concentrations of the main acetaldehyde precursors, and 5 times the precursors would bring the feedback to 0.17 ppbv h^–1^. The effect of primary acetaldehyde on secondary acetaldehyde formation would increase from −0.014 to 0.11 ppbv h^–1^ if the NO concentration is 10 times higher. It is noteworthy that 5 times the precursors (e.g., 0.5 ppbv of *trans*-2-butene) and 10 times the NO (4.1 ppbv) were not high relative to their common concentrations in urban areas. Therefore, we expect that some of the above interactions, if not all, would be significant in polluted air. Since the primary OVOCs and O_3_ facilitate secondary OVOC formation through replenishing oxidative radicals, holistic management of atmospheric oxidative capacity would likely weaken the positive feedbacks between OVOCs and O_3_.

## 4 Atmospheric Implications

It has been well documented that biogenic emissions play an important role in regulating atmospheric chemistry. The role becomes increasingly significant under warming climate, forming an undesirable synergy with climate hazards. For example, heatwaves may witness O_3_ pollution primarily driven by enhanced biogenic emissions. However, studies on biogenic emissions have mainly focused on nonmethane hydrocarbons such as isoprene and monoterpenes and paid little attention to OVOCs. This study demonstrated significant biogenic emissions and/or formation of several OVOCs, spanning from the least reactive acetone to the most reactive acrolein, which accounted for more than half of the observed concentrations except for ethanol. Therefore, it is essential to fully characterize biogenic emissions of OVOCs for dominant vegetation species under typical atmospheric conditions and, if applicable, establish the relationships with common biogenic VOCs, e.g., isoprene. Moreover, the chemical mechanisms of biogenic VOC degradation and OVOC formation should be revisited and upgraded where necessary. Proper representation of OVOC emissions and chemistry has the potential to enhance our understanding of how OVOCs impact photochemistry. At this background site, the impacts appeared to be more related to the O_3_ formation sensitivity to the precursors. Although it is unlikely to apply in all scenarios, this provides a perspective that O_3_ pollution driven by biogenic emissions could be mitigated through reducing anthropogenic emissions, e.g., NO_*x*_. However, the presence of biogenic OVOCs may make it necessary to increase the reduction of anthropogenic emissions. Lastly, understanding the complex OVOC–O_3_ interactions, as revealed for acetaldehyde in this study, will aid in the development and evaluation of the O_3_ control strategies. Despite the above implications, the findings for this subset of OVOCs do not necessarily apply to all OVOCs in the air.

## Supporting Information Available

Selection of OVOCs; isoprene correction; initial X/B ratio calculation; determination of coefficient *m*; site location; comparison of *m*/*z* 69 and isoprene; model validation; formaldehyde concentrations; diurnal patterns of OVOCs; diurnal patterns of anthropogenic primary and secondary OVOCs; correlations of OVOCs with tracers; in situ formation and loss of acetaldehyde; measurements used in this study; quality control metrics; X/B ratios; model input; fitted values of key parameters; biogenic contributions; and precursors and formation pathways of acetaldehyde (PDF)
