# Supplementary material for: Significant Biogenic Source of Oxygenated Volatile Organic Compounds and the Impacts on Photochemistry at a Regional Background Site in South China
Source: Environ Sci Technol. 2024 Nov 1;58(45):20081–90. doi: 10.1021/acs.est.4c05656 (PMC11562714; doi:10.1021/acs.est.4c05656)
Supplement: Supplementary file 1 — es4c05656_si_001.pdf [file es4c05656_si_001.pdf]

*Supporting Information for*

# Significant biogenic source of oxygenated volatile organic compounds and the impacts on photochemistry at a regional background site in South China

Xiaopu Lyu, Hongyong Li, Shun-cheng Lee, Enyu Xiong, Hai Guo, Tao Wang, Joost de Gouw

Correspondence to xiaopu_lyu@hkbu.edu.hk

This 13-page document contains 4 texts, 8 figures, and 7 tables.

## Supplementary Texts

**Text S1.** Selection of OVOC species

In this sampling campaign, dozens of OVOCs were measured using the proton transfer reaction mass spectrometry technique. As introduced in a previous study,[1] the measurements of 6 OVOC species (acetaldehyde, acrolein, acetone, 2-propanol, methyl ethyl ketone, benzaldehyde) were calibrated using a mixture of authentic standards consisting of the above OVOCs and 6 non-methane hydrocarbons. Empirical reaction kinetics were adopted to determine the sensitivity for the uncalibrated species.[2] In this study, many OVOCs were excluded from the analysis for the following reasons. First, the species with low data quality were eliminated. Second, it was difficult to identify some molecules accurately. For example, the identification of methyl acetate at m/z 75.044 ($C_3H_6O_2H^+$) might be interfered by propionic acid and ethyl formate. Third, the concentrations of many OVOCs were low, such as 0.037 ppbv for benzaldehyde. Fourth, a parallel study used the data of more OVOCs, and we intentionally avoided duplication of content. More importantly, the 6 OVOCs we focused on were representative. For example, methyl ethyl ketone and acetic acid correlated well with acetaldehyde, with the $R^2$ of 0.80 and 0.81, respectively. The rate constant for the reactions between the selected OVOCs with OH varied in a wide range from $1.7\times10^{-13}$ $cm^3$ $molecule^{-1}$ $s^{-1}$ for acetone to $2\times10^{-11}$ $cm^3$ $molecule^{-1}$ $s^{-1}$ for acrolein, suggesting their broad representation in atmospheric reactivity.

**Text S2.** Correction of PTR-measured isoprene

The m/z 69 signal measured by PTR was corrected using the method illustrated in a recent study.[3] The ratio of m/z 69 to m/z 111 (f69/111) during 4:00-6:00 local time (LT) was determined as 3.76, based on the least-square linear regression between the two ions (R = 0.88). This time period was chosen because the temperature was the lowest and we expect biogenic emissions to be as well. The corrected isoprene was calculated using the formula below.

$$\text{Isoprene} = \text{m/z } 69 - f69/111 \times m/z\ 111$$

**Text S3.** Calculation of $\frac{[xylenes]}{[benzene]}_{t=0}$

The hourly molar ratio of xylenes to benzene (X/B) was calculated for all the samples. According to the average diurnal profile, the ratio was the highest during 18:00-0:00, indicating the freshest air masses in this time period. The data of xylenes and benzene corresponding to this subset of the X/B ratio during 18:00-0:00 was selected, based on which we performed a linear fit between the natural log of xylenes and benzene. With the equation shown in Figure 1a, we calculated the fitted mixing ratio of xylenes at the point where the observed mixing ratio of benzene was the highest among all the samples. The

ratio of the fitted xylenes to the observed benzene was regarded as the initial X/B ratio $\frac{[xylenes]}{[benzene]}_{t=0}$.

**Text S4.** Determination of the coefficient m in formula (1)

The photochemical-age-based parameterization method only considers the chemical loss of OVOCs through reacting with OH, but this is not the case for some OVOC species, due to the presence of significant photolysis. To correct for the chemical loss, a coefficient *m* is introduced into the formula (1). For a specific OVOC species, the coefficient *m* was calculated as the ratio of total reaction rate of chemical loss (mainly photolysis and OH-initiated oxidation) to the reaction rate of OH-initiated oxidation. The ratio in the air mass history was considered the same as that simulated at the HT site (in-situ photochemistry), as elucidated in sections 2.3 & 3.3.

## Supplementary Figures

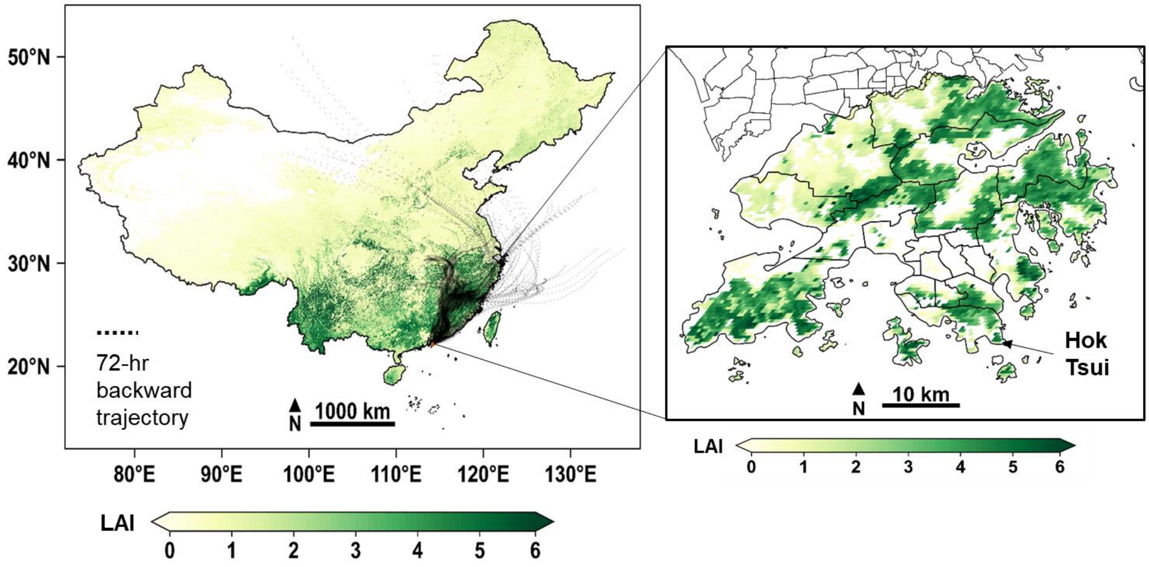


**Figure S1.** Location of the sampling site and 72-hr backward trajectories reaching 300 m above the site. The background map is color-coded by leaf area index (LAI) in autumn 2020. LAI data is download from https://lpdaac.usgs.gov/products/mod15a2hv061/.

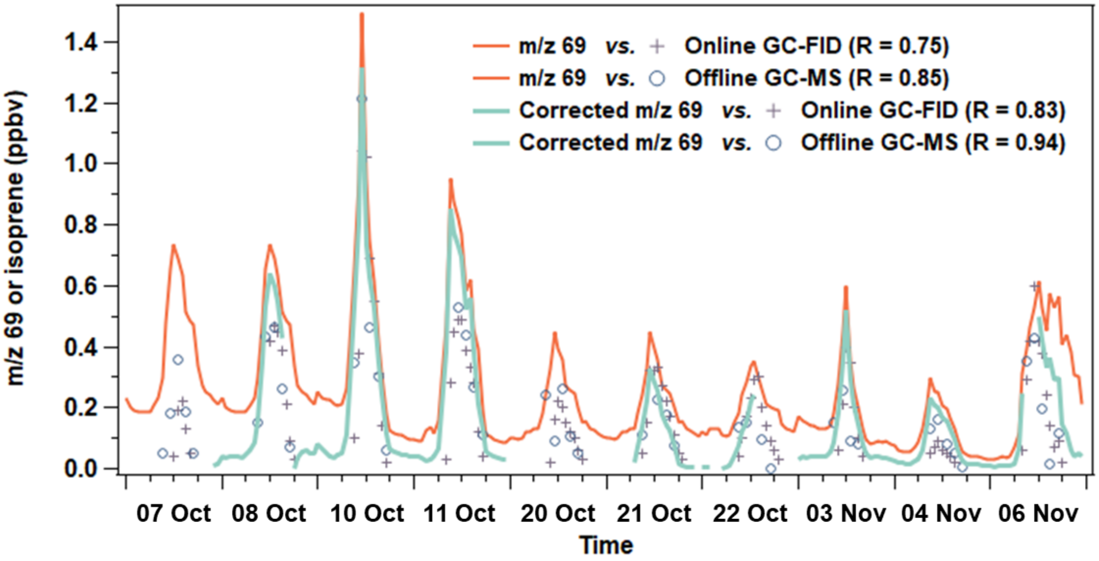


**Figure S2.** Time series of m/z 69, corrected m/z 69 and isoprene measured by online and offline GC systems on the 10 selected dates. R represents the correlation coefficient between any two sets of data.

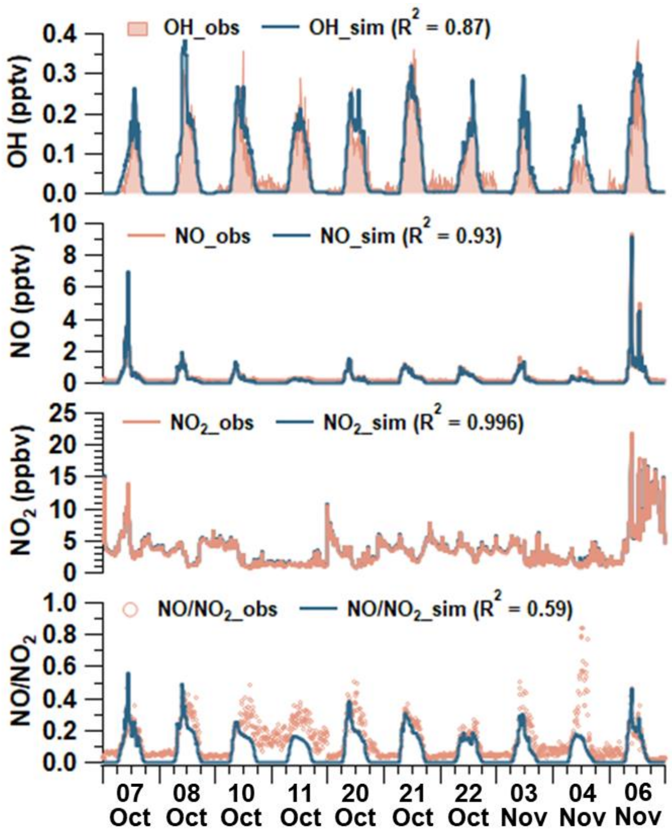


**Figure S3.** Time series of the simulated and observed OH, NO, $NO_2$ and the NO/$NO_2$ ratio (10-min data).

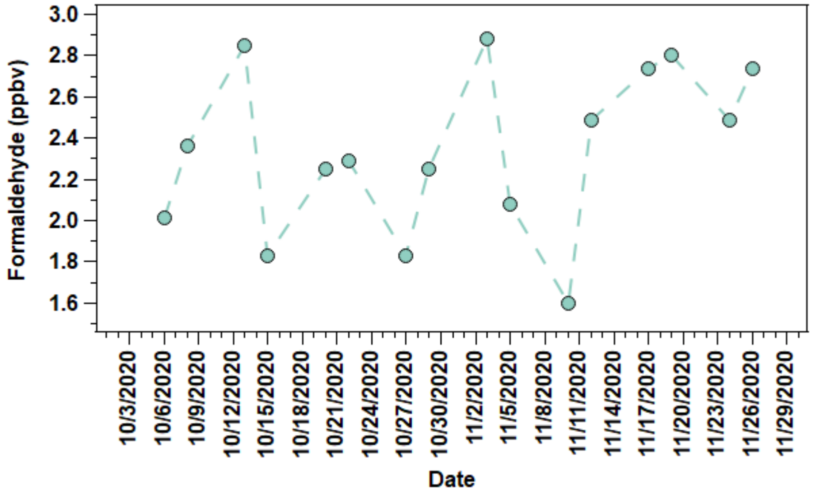


**Figure S4.** Variations in daily average concentration of formaldehyde at the HT site.

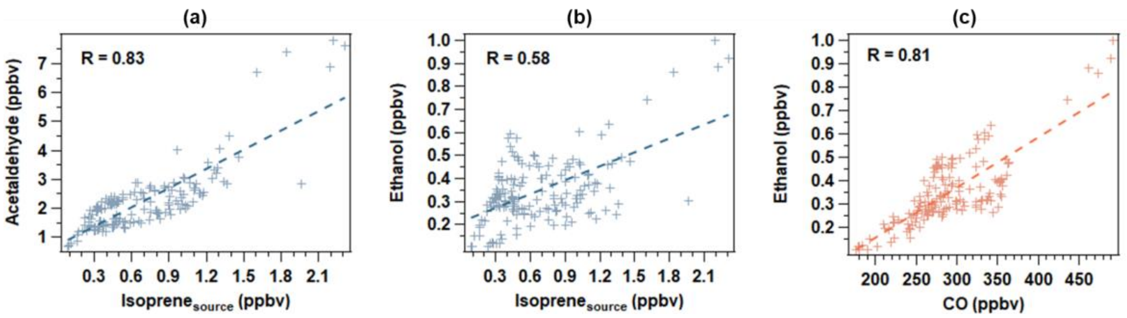


**Figure S5.** Correlations between acetaldehyde and $isoprene\_source$ (a); ethanol and $isoprene\_source$ (b); and ethanol and CO (c).

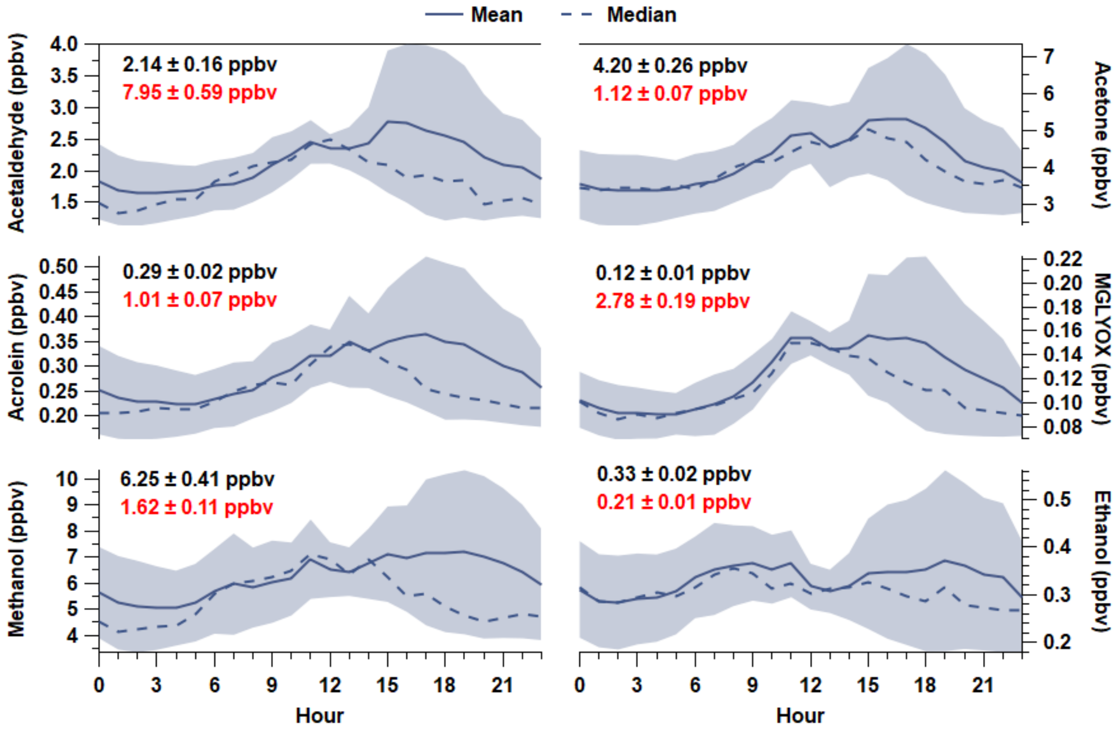


**Figure S6.** Diurnal patterns of OVOCs represented with mean and median values. Black and red fonts indicate average concentrations and OFPs of OVOCs, respectively.

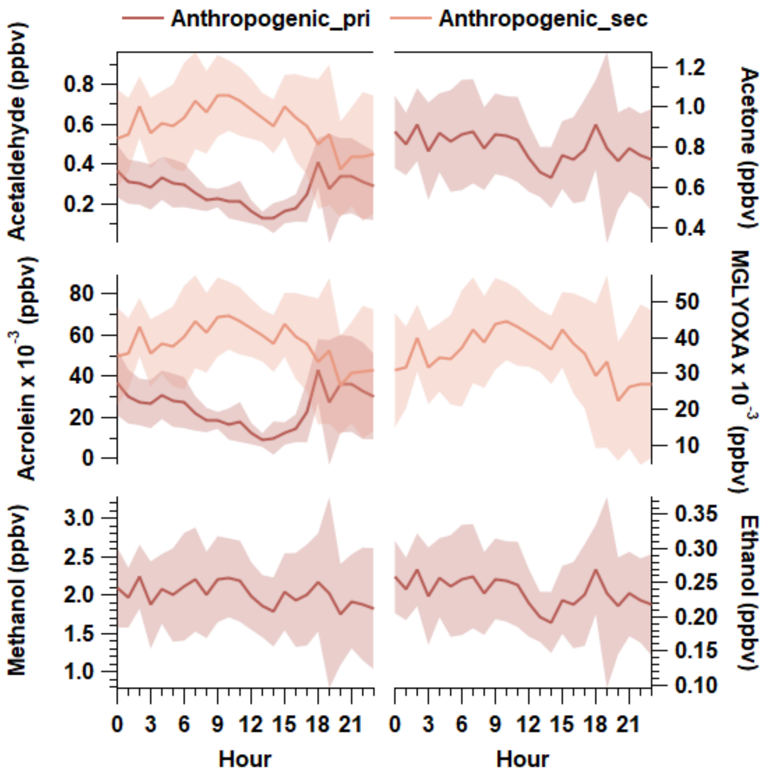


**Figure S7.** Average diurnal patterns of anthropogenic primary and anthropogenic secondary OVOCs determined by the photochemical-age-dependent parameterization method.

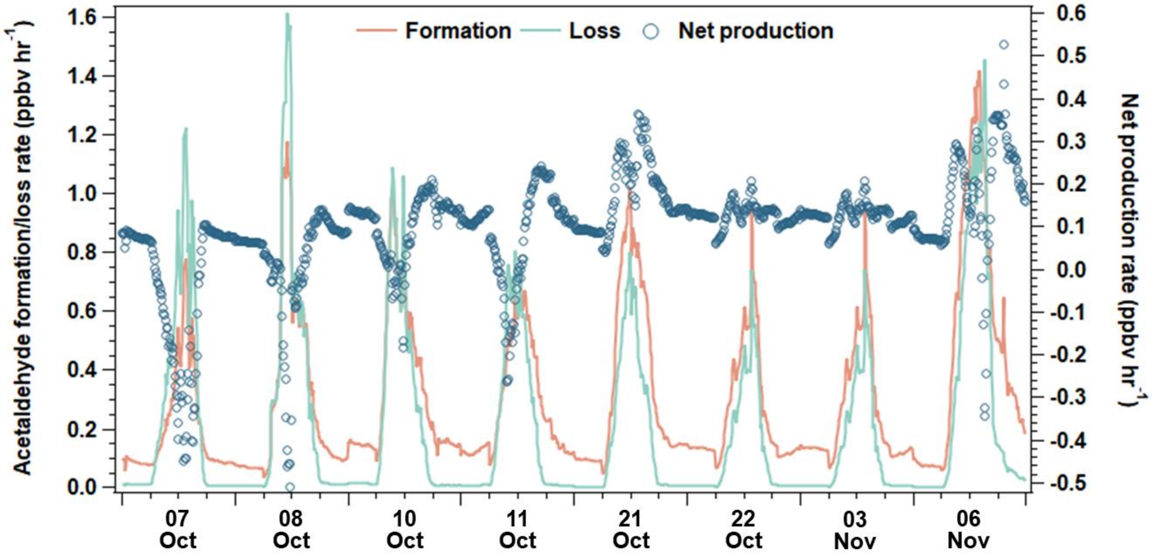


**Figure S8.** In-situ formation, loss and net production rate of acetaldehyde simulated with the F0AM model.

## Supplementary Tables

**Table S1.** Measurements of the key species/parameters and their main uses in this study.

| **Species / Parameters** | **Instrument** | **Main uses in this study** |
|---|---|---|
| OVOCs | Ionicon Proton Transfer Reaction quadrupole ion Time-of-Flight Mass Spectrometer (PTR) | • Main research objects |
| Non-methane hydrocarbons (online hourly data) | Gas Chromatography – Mass Spectrometry / Flame Ionization Detection | • Xylenes and benzene for photochemical age estimation<br>• Benzene as the anthropogenic tracer<br>• Isoprene to validate PTR isoprene correction |
| Non-methane VOCs (offline data) | Gas Chromatography – Mass Spectrometry / Flame Ionization Detection / Electron Capture Detection | • F0AM model input<br>• Isoprene to validate PTR isoprene correction |
| Nitrous acid (HONO) | Long-Path-Absorption-Photometer (LOPAP) | • F0AM model input |
| OH radical | Chemical Ionization Mass Spectrometer | • Model validation |
| Trace gases, including $O_3$, nitric oxide (NO), nitrogen dioxide ($NO_2$), carbon monoxide (CO), sulfur dioxide ($SO_2$) | Thermo trace gas analyzers | • F0AM model input<br>• NO and $NO_2$ for model validation<br>• CO as anthropogenic tracer |
| Photolysis frequency of nitrogen dioxide ($jNO_2$) | MetCon Filter Radiometer | • F0AM model input |
| Meteorological parameters | Weather station operated by Hong Kong Observatory (HKO) | • F0AM model input |

**Table S2.** Metrics of data quality control for VOCs and OVOCs used in this study.

| Species | Analysis technique | Accuracy | Precision | Detection limit (pptv) |
|---|---|---|---|---|
| Acetaldehyde | PTR | -* | -* | 2# |
| Acetone | PTR | - | - | 8 |
| Acrolein | PTR | - | - | 8 |
| Methylglyoxal | PTR | - | - | 11 |
| Methanol | PTR | - | - | 30 |

| | | | | |
|---|---|---|---|---|
| Ethanol | PTR | - | - | 18 |
| Isoprene | PTR | - | - | 25 |
| Ethane | GC-FID | 7.2% | 9.9% | 20 |
| Propane | GC-FID | 9.9% | 10.2% | 14 |
| *i*-Butane | GC-MSD | 1.3% | 3.3% | 28 |
| *n*-Butane | GC-MSD | 5.4% | 3.2% | 33 |
| *i*-Pentane | GC-MSD | 8.2% | 4.5% | 18 |
| *n*-Pentane | GC-MSD | 2.1% | 4.3% | 32 |
| 2,2-Dimethylbutane | GC-MSD | 3.3% | 8.1% | 18 |
| 2,3-Dimethylbutane | GC-MSD | 5.5% | 4.5% | 15 |
| 2-Methylpentane | GC-MSD | 7.8% | 3.9% | 19 |
| 3-Methylpentane | GC-MSD | 4.8% | 5.9% | 21 |
| *n*-Hexane | GC-MSD | 4.4% | 8.0% | 14 |
| Cyclohexane | GC-MSD | 4.5% | 8.1% | 10 |
| 2-Methylhexane | GC-MSD | 2.7% | 6.4% | 19 |
| 3-Methylhexane | GC-MSD | 3.2% | 7.4% | 28 |
| *n*-Heptane | GC-MSD | 3.4% | 9.3% | 11 |
| *n*-Octane | GC-MSD | 2.3% | 8.1% | 19 |
| *n*-Nonane | GC-MSD | 0.4% | 6.8% | 14 |
| *n*-Decane | GC-MSD | 4.6% | 12.9% | 5 |
| Ethene | GC-FID | 7.8% | 10.2% | 8 |
| Propene | GC-FID | 7.1% | 10.3% | 12 |
| 1-Butene | GC-MSD | 1.6% | 18.5% | 26 |
| 1,3-Butadiene | GC-MSD | 9.7% | 5.2% | 0.3 |
| *Trans*-2-butene | GC-MSD | 4.3% | 2.1% | 30 |
| *Cis*-2-butene | GC-MSD | 3.7% | 4.4% | 23 |
| 1-Pentene | GC-MSD | 6.1% | 4.4% | 35 |
| Isoprene | GC-MSD | 18.3% | 15.1% | 8 |
| Acetylene | GC-FID | 6.8% | 10.1% | 14 |
| Benzene | GC-MSD | 2.7% | 6.8% | 20 |
| Toluene | GC-MSD | 1.6% | 7.7% | 13 |
| Ethylbenzene | GC-MSD | 0.1% | 5.9% | 5 |
| *m,p*-Xylene | GC-MSD | 1.1% | 6.1% | 20 |
| *o*-Xylene | GC-MSD | 1.2% | 5.8% | 6 |
| Styrene | GC-MSD | 2.2% | 7.7% | 2 |
| *i*-propylbenzene | GC-MSD | 1.1% | 5.3% | 12 |
| *n*-propylbenzene | GC-MSD | 5.6% | 8.7% | 6 |
| 3-ethyltoluene | GC-MSD | 7.2% | 13.8% | 15 |
| 4-ethyltoluene | GC-MSD | 8.3% | 12.2% | 19 |
| *o*-ethyltoluene | GC-MSD | 5.3% | 11.4% | 18 |
| 1,3,5-trimethylbenzene | GC-MSD | 4.4% | 9.2% | 13 |
| 1,2,4-trimethylbenzene | GC-MSD | 7.0% | 12.5% | 16 |
| 1,2,3-trimethylbenzene | GC-MSD | 8.3% | 16.7% | 10 |
| VOC data provided by the EPD † | GC-MSD/FID | 1.0-10.0% | 2.5-20.0% | 2-787 |

* Accuracy and precision are not determined for PTR measurements. The sensitivity ($R^2$ of response curve) is 164.6 cps/ppbv (0.990), 501.6 cps/ppbv (0.997) and 476.5 cps/ppbv (0.990) for the measurement of acetaldehyde, acetone and acrolein, respectively.

# Detection limit of PTR measurements for 1s integration.

† 29 VOC species including benzene, *m,p*-xylenes and isoprene that are used in this study.

**Table S3.** Ratio of *m,p*-xylenes to benzene measured in a tunnel and at five roadside sites in HK during selected periods.

| Measurement location | Study period | Data type | Benzene | *m,p*-Xylenes | X/B ratio (ppbv/ppbv) |
|---|---|---|---|---|---|
| Shing Mun Tunnel, HK* | Summer & winter 2003 | Emission factor | 4.5 ± 0.9 mg $vehicle^{-1}$ $km^{-1}$ | 3.7 ± 0.7 mg $vehicle^{-1}$ $km^{-1}$ | 0.61 ± 0.17 |
| Five roadside sites in HK# | 10:00am, Sep 2013 – Jun 2014 | Ambient mixing ratio | 676 ± 61 pptv | 494 ± 76 pptv | 0.73 ± 0.13 |

* Data from Ho et al.[4]; # unpublished data shared by the EPD.

**Table S4.** Species and parameters that are input to constrain the F0AM model.

| Data category | Name | Data source |
|---|---|---|
| Trace gases | $O_3$ | Regular measurements by the EPD |
| | $NO_x$ | |
| | CO | |
| | $SO_2$ | |
| VOCs | 18 alkanes | Analysis results of canister samples |
| | 8 alkenes | |
| | Acetylene | |
| | 14 aromatics | |
| OVOCs | Acetaldehyde | PTR measurements in this field campaign |
| | Acetone | |
| | Acrolein | |
| | Methylglyoxal | |
| | Methanol | |
| | Ethanol | |
| HONO | HONO | LOPAP measurement in this field campaign |
| Meteorological parameters | $jNO_2$ | MetCon Filter Radiometer measurement in this field campaign |

| | | |
|---|---|---|
| | Temperature | Regular measurements by the HKO |
| | Relative humidity | |

**Table S5.** Fitted values with standard deviations for the key parameters (except the known $k_{ovoc}$) in the parameterization method.

| OVOC species | $k_{ovoc}$ ($10^{-12}$ $cm^3$ $molecule^{-1}$ $s^{-1}$) [a] | $k_{precursor}$ ($10^{-12}$ $cm^3$ $molecule^{-1}$ $s^{-1}$) | $ER_{OVOC}$ (ppbv/ppbv benzene) | $ER_{precursor}$ (ppbv/ppbv benzene) | $ER_{biogenic}$ (ppbv/ppbv $isoprene_{source}$) | Background (ppbv) |
|---|---|---|---|---|---|---|
| Acetaldehyde | 15 | 5.81 ± 3.16 | 3.64 ± 1.17 | 14.7 ± 4.28 | 2.03 ± 0.09 | 0 |
| Acetone | 0.17 | 0 | 4.67 ± 1.25 | 0 | 3.61 ± 0.18 | 1.25 ± 0.25 |
| Acrolein | 20 | 3.16 ± 3.82 | 0.45 ± 0.19 | 2.61 ± 2.34 | 0.28 ± 0.01 | 0.03 ± 0.02 |
| MGLYOX | 15 | 1.58 ± 1.29 | 0 | 6.05 ± 4.12 | 0.11 ± 0.01 | 0.005 ± 0.007 |
| Methanol | 0.94 | 0 | 9.91 ± 1.65 | 0 | 4.96 ± 0.30 | 0.99 ± 0.40 |
| Ethanol | 3.2 | 0 | 1.38 ± 0.06 | 0 | 0.17 ± 0.01 | 0 |

[a] Data from MCM v3.3.1 and Atkinson and Arey (2003).[5]

**Table S6.** Fitted biogenic contributions to MACR/MVK and the other OVOCs studied.

| | R [*] | Biogenic contribution (%) |
|---|---|---|
| MACR/MVK | 0.95 | 88.7 |
| Acetaldehyde [†] | 0.84 | 47.7 |
| Acetone [†] | 0.73 | 37.8 |
| Acrolein [†] | 0.75 | 56.0 |
| MGLYOX [†] | 0.81 | 41.9 |
| Methanol [†] | 0.70 | 48.6 |
| Ethanol [†] | 0.74 | 25.6 |

[*] Correlation coefficient between the fitted and observed values; [†] Data on Nov 6 is excluded from the regression.

**Table S7.** Main reactions leading to acetaldehyde formation, corresponding VOC precursors and contributions to acetaldehyde formation rate at 12:00 LT. Reactions initiated by acetaldehyde + OH are excluded.

| Last reaction | Main initial reaction | Precursor | Daytime average acetaldehyde formation rate (ppbv $hr^{-1}$) | Cumulative contribution (%) |
|---|---|---|---|---|
| BUT2OLAO = CH3CHO + | cis-/trans-2-butuene + OH | cis-/trans-2-butuene | 0.129 | 30.4 |

| | | | | |
|---|---|---|---|---|
| CH3CHO + HO2 | | | | |
| TBUT2ENE + O3 = CH3CHO + CH3CHOOB | cis-/trans-2-butuene + $O_3$ | cis-/trans-2-butuene | 0.096 | 53.1 |
| CBUT2ENE + O3 = CH3CHO + CH3CHOOB | cis-/trans-2-butuene + $O_3$ | cis-/trans-2-butuene | 0.054 | 65.8 |
| HYPROPO = CH3CHO + HCHO + HO2 | propene + OH | propene | 0.032 | 73.3 |
| C23O3MO = CH3CHO + CH3CO3 | trimethylbenzene + OH | trimethylbenzene | 0.014 | 76.7% |
| C2H5OH + OH = CH3CHO + HO2 | ethanol + OH | ethanol | 0.013 | 79.7% |
| C2H5O = CH3CHO + HO2 | *i*-pentane + OH | *i*-pentane | 0.011 | 82.3% |
| CH3CHOO = CH3CHO + H2O2 | cis-/trans-2-butuene + $O_3$ | cis-/trans-2-butuene | 0.010 | 84.7% |
| MEKBO = CH3CHO + CH3CO3 | cis-/trans-2-butuene + $NO_3$ | cis-/trans-2-butuene | 0.010 | 87.0% |
| MEKBO = CH3CHO + CH3CO3 | trimethylbenzene + OH | trimethylbenzene | 0.008 | 88.9% |
| C2H5O = CH3CHO + HO2 | *n*-butane + OH | *n*-butane | 0.006 | 90.3% |
| IPROPOLO = CH3CHO + HCHO + HO2 | propene + OH | propene | 0.005 | 91.5% |
| SC4H9O = CH3CHO + C2H5O2 | *n*-butane + OH | *n*-butane | 0.005 | 92.7 |
| O3 + C3H6 = CH2OOB + CH3CHO | propene + $O_3$ | propene | 0.003 | 93.4 |

| | | | | |
|---|---|---|---|---|
| C92O = CH3CHO + HO5C6CO3 | *n*-nonane + OH | *n*-nonane | 0.003 | 94.1 |
| C42NO33O = CH3CHO + CH3CHO + NO2 | cis-/trans-2-butuene + $NO_3$ | cis-/trans-2-butuene | 0.003 | 94.8 |
